# Impact of Respiratory Support Strategies on CO_2_ Variability During Therapeutic Hypothermia in Neonates With Asphyxia: Data From the German Hypothermia Registry

**DOI:** 10.1111/apa.70702

**Published:** 2026-07-17

**Authors:** Sebiha Demir, Anne Groteklaes, Bettina Bohnhorst, Kirsten Glaser, Andreas Müller, Hemmen Sabir, Till Dresbach, the German Hypothermia Registry, Milian Brasche, Nicolas Allgaier, Daniel Klotz, Norbert Teig, Katja Schneider, Hemmen Sabir, Sandra Holz, Birte Tröger, Thorsten Körner, Uwe Höpner, Marie Schmenk, Michael Hofmann, Mario Rüdiger, Julia Osterholt, Martin Berghäuser, Christopher Willerscheidt, Farina Morgenroth, Hans Jörg Bittrich, Steven Hébert, Arber Arapi, Anja Stein, Veit Lange, Gunnar Rau, Lisa‐Marie Stoek, Nikolas Hillenbrand, Matthias Reger, Susanne Schade, Jana Dieks, Heimke von Osten, Marita Horstkemper, Martin Blohm, Mario Lange, Bettina Bohnhorst, Manuela Friederike Richter, Simon Kranig, Maurice Petrasch, Nasenien Nourkami‐Tutdibi, Christine Pinkwart, Thomas Kriebel, Silvia Welcker, Nora Bonney, Angela Kribs, Clemens Andrée, Sophia Stadermann, Kirsten Glaser, Peter Jahn, Katrin Braun, Anna Eisenhardt, Alba Perez‐Ortiz, Nadine Mand, Ines Wallot, Timea Takacs, Anja Jäger, Oliver Schroers, Esther Schouten, Claudia Nussbaum, Kai Böckenholt, Rüdiger Wentzell, Guido Engelmann, Katherine Louise Kastor, Michael Schroth, Matthias Engler, Friederike Eichhorn, Moritz Rohrbach, Fabian Bärtling, Frank Dohle, Matthias Keller, Marion Weber, Verena Lehnerer, Anja Mayer, Marie‐Claire Detemple, Valentin Oellers, Markus Pingel, Friederike Eilbracht, Jutta Adler, Elena Eberle, Karoline Goj, Virginia Toth, Andrea Czoske, Cornelia Wiechers, Christiane Mayer, Anja Schumann, Patrick Salaschek, Janaina Rauch, Kai Hensel, Pia Paul

**Affiliations:** ^1^ Department of Neonatology and Pediatric Intensive Care Children's Hospital, University of Bonn Bonn Germany; ^2^ Department of Pediatric Pneumology, Allergology and Neonatology Hannover Medical School Hannover Germany; ^3^ Division of Neonatology, Department of Women's and Children's Health University of Leipzig Medical Center Leipzig Germany; ^4^ German Center for Neurodegenerative Diseases (DZNE) Bonn Germany

**Keywords:** carbon dioxide, hypoxic–ischaemic encephalopathy, perinatal asphyxia, respiratory support, therapeutic hypothermia

## Abstract

**Aim:**

To evaluate associations between respiratory support strategies, partial pressure of carbon dioxide (pCO_2_) variability and oxygen exposure in neonates with hypoxic–ischaemic encephalopathy (HIE) undergoing therapeutic hypothermia (TH).

**Methods:**

This retrospective multicentre study used data from the German Hypothermia Registry, including neonates ≥ 36 weeks with moderate‐to‐severe HIE treated with TH between 2022 and 2025. Respiratory support was categorized as continuous positive airway pressure (CPAP) or invasive ventilation. Maximum and minimum pCO_2_ and fraction of inspired oxygen (FiO_2_) were analysed during the first 6 and 72 h of life.

**Results:**

Among 428 neonates, 99% required respiratory support and 88% received invasive ventilation. Ventilated neonates showed higher maximum pCO_2_ levels than CPAP‐supported neonates within the first 6 h (70 vs. 55 mmHg, *p* = 0.005) and over 72 h (70 vs. 60 mmHg, *p* < 0.001), while minimum pCO_2_ values were lower. Invasive ventilation was also associated with higher FiO_2_ requirements and longer oxygen supplementation (4 vs. 1 days, *p* < 0.001). In neonates with moderate HIE, CPAP was associated with shorter oxygen exposure, earlier oral feeding and reduced hospital stay.

**Conclusion:**

Respiratory support strategy during TH is associated with distinct patterns of CO_2_ and oxygen exposure. CPAP appears feasible in selected neonates with moderate HIE and favourable amplitude‐integrated electroencephalography (aEEG) patterns.

## Summary

- Respiratory management during therapeutic hypothermia for hypoxic–ischaemic encephalopathy (HIE) remains poorly standardized.
- Invasive ventilation was associated with greater carbon dioxide variability and longer oxygen supplementation compared with continuous positive airway pressure (CPAP).
- CPAP appeared feasible in selected neonates with moderate HIE and favourable amplitude‐integrated electroencephalography patterns and was associated with improved short‐term clinical outcomes.

## 1 Introduction

Therapeutic hypothermia (TH) is the standard neuroprotective treatment for term and near‐term neonates with moderate to severe hypoxic–ischaemic encephalopathy (HIE) following perinatal asphyxia [1, 2]. Although TH has improved survival and neurodevelopmental outcomes, a substantial proportion of affected neonates continue to experience death or long‐term neurological impairment [3, 4]. This highlights the importance of optimizing supportive intensive care during TH to prevent secondary brain injury [5].

Respiratory management is a key component of supportive care during TH, yet remains insufficiently standardized [6, 7]. This is clinically relevant because most neonates enrolled in randomized controlled trials and large observational studies of TH were mechanically ventilated [1], often reflecting the severity of encephalopathy, the need for sedation, or concerns regarding respiratory instability during cooling. Moreover, it is often assumed that sedation and mechanical ventilation attenuate the secondary brain injury by reducing physiological stress, suppressing neuronal activity, stabilizing oxygenation and carbon dioxide levels and decreasing systemic and cerebral energy expenditure.

In patients with HIE, hypocapnia is one of the main risk factors for secondary brain injury. Carbon dioxide (CO_2_) plays a central role in the regulation of cerebral blood flow, with even small deviations in arterial CO_2_ leading to rapid changes in cerebrovascular tone [8]. Hypocapnia induces cerebral vasoconstriction and may reduce cerebral perfusion, whereas hypercapnia causes vasodilation and may increase intracranial blood volume [6]. Because cerebral autoregulation is frequently impaired in neonates with HIE, fluctuations in CO_2_ may substantially alter blood flow and contribute to secondary brain injury [7, 8]. Both hypo‐ and hypercapnia have been associated with adverse neurological outcomes in neonates with HIE [9]. Observational studies have linked early hypocapnia to increased mortality, abnormal neuroimaging findings and poorer neurodevelopmental outcomes [10]. Conversely, excessive hypercapnia may exacerbate cerebral edema and increase intracranial pressure, potentially aggravating secondary brain injury [11]. Despite these concerns, optimal CO_2_ target ranges during TH have not been clearly defined. TH further complicates respiratory management by reducing metabolic rate and CO_2_ production, increasing the risk of hypocapnia, particularly in spontaneously breathing infants [6, 7]. In addition, interpretation of blood gas values during hypothermia is influenced by temperature‐dependent changes in gas solubility and clinical practice regarding temperature correction remains variable [8, 9]. While invasive mechanical ventilation is commonly employed during TH, non‐invasive respiratory support such as continuous positive airway pressure (CPAP) is increasingly used in clinically stable neonates. Multicentre data on respiratory management during TH are limited, particularly with respect to CO_2_ variability and oxygen exposures across different modes of respiratory support [10].

In addition to carbon dioxide, oxygen exposure plays a critical role in cerebral pathophysiology following perinatal asphyxia [11, 12, 13]. Both hypoxia and hyperoxia may contribute to secondary brain injury [11]. During the reperfusion phase after hypoxic–ischaemic injury, excessive oxygen delivery can increase the generation of reactive oxygen species, amplifying oxidative stress, mitochondrial dysfunction and inflammatory responses in the vulnerable neonatal brain [12, 13]. Experimental and clinical studies have demonstrated associations between early hyperoxia and worse neurological outcomes following perinatal asphyxia [11, 12]. TH modifies cerebral oxygen consumption and systemic oxygen requirements, further complicating oxygen titration [11, 13]. Despite recommendations to avoid both hypoxia and hyperoxia, optimal oxygen targets during TH remain poorly defined and real‐world data on oxygen exposure in cooled neonates are scarce [8, 14].

Recently, the German Hypothermia Registry has been established, collecting data from cooled asphyxiated newborns from over 80 German neonatal units. The registry was established in 2022, with patient enrolment beginning in the same year. Using data from the German Hypothermia Registry, this study aimed to characterize respiratory management strategies in neonates with moderate to severe HIE undergoing TH and to assess whether the mode of respiratory support (invasive ventilation vs. CPAP) is associated with differences in oxygen exposure and CO_2_ instability.

## 2 Methods

### 2.1 Study Design and Population

This study is a retrospective registry‐based analysis using data from the German Hypothermia Registry, a multicentre national registry that collects detailed clinical data on neonates treated with TH in Germany. Inclusion criteria were term and near‐term neonates (≥ 36 weeks gestation) with moderate or severe HIE who received whole‐body TH between December 2022 and December 2025. During this period, the registry included a total of 429 neonates treated in 80 neonatal intensive care units (NICUs) across Germany.

The registry collects data in accordance with international guideline recommendations for the management of neonates with HIE undergoing TH [15]. For participating centres of the German Hypothermia Registry, a standardized treatment protocol for TH is in place, which also includes potential recommendations for respiratory management. The protocol aims for near‐normal oxygenation and ventilation, targeting PaO_2_ values of 50–100 mmHg and PaCO_2_ values of 40–50 mmHg while using the lowest possible fraction of inspired oxygen (FiO_2_). In spontaneously breathing neonates, hypocapnia in the context of compensatory tachypnea due to metabolic acidosis is tolerated, whereas in invasively ventilated patients, hypercapnia and hyperoxia should be avoided; continuous CO_2_ monitoring is recommended if applicable [16].

### 2.2 Ethics Approval

The study was approved by the Ethics Committee of the University of Bonn (approval number: 472/22‐EP). As this study involved retrospective analysis of fully anonymized patient data, individual informed consent from parents or legal guardians was waived by the Ethics Committee.

### 2.3 Data Collection

Data were collected retrospectively from the German Hypothermia Registry using a standardized electronic report form implemented in REDCap (Research Electronic Data Capture), a secure, web‐based data capture system. The following variables were collected for each patient:

- Demographics and clinical characteristics: gestational age, birth weight, Apgar scores at 1, 5 and 10 min, initial pH, resuscitation, HIE grade and amplitude‐integrated electroencephalography (aEEG) background activity (classified as continuous normal voltage, discontinuous normal voltage, burst suppression, low voltage or flat trace. Documentation of electrical seizures). Resuscitation at birth was defined as the requirement for interventions beyond routine postnatal care, such as positive pressure ventilation, intubation, chest compressions, or medication.
- Respiratory support: initial ventilation mode (CPAP or invasive mechanical ventilation), timing of endotracheal intubation (before, during, or after initiation of TH) and total duration of respiratory support, as well as the use of sedative and/or analgesic medication, including whether sedation was applied and the specific agents used.
- Respiratory parameters: FiO_2_, partial pressure of carbon dioxide (pCO_2_)

Respiratory parameters were analysed during two predefined periods: the first 6 h of life and the first 72 h after birth. Within each period, maximum and minimum values of FiO_2_ and pCO_2_ were recorded. Additionally, the duration of supplemental oxygen therapy was documented.

Due to the anonymised nature of data collection within the registry, no additional ventilatory‐associated data was collected. Neonates with major congenital anomalies, chromosomal abnormalities, or missing respiratory data were excluded.

### 2.4 Statistical Analysis

Continuous variables are presented as medians with interquartile ranges (IQR) and categorical variables are presented as frequencies and percentages. Group comparisons for categorical variables, such as timing of intubation, were performed using chi‐square tests. Differences in continuous variables between two groups, including FiO_2_, duration of oxygen supplementation and pCO_2_ values, were analysed using the Mann–Whitney U test. For analyses over time, respiratory parameters were assessed in two predefined periods: the first 6 h of life (early stabilization) and the first 72 h after birth (during TH). Maximum and minimum pCO_2_ values were extracted for each period and compared between ventilation modes (CPAP vs. invasive mechanical ventilation). A *p* value < 0.05 was considered statistically significant. All statistical analyses were performed using SPSS version 29 (IBM Corp, New York, USA).

## 3 Results

A total of 429 neonates with moderate to severe HIE treated with TH were included in the analysis, and one patient was excluded due to missing respiratory data. Baseline characteristics of the cohort are summarized in Table 1. A total of 425 neonates (99%) needed any kind of respiratory support after birth. At initiation of support, 246 neonates (57%) were managed using CPAP, while 170 (40%) were primarily invasively ventilated following birth. Among neonates who required resuscitation at birth (*n* = 97), 63 (65%) were immediately intubated. Overall, 375 neonates (88%) required invasive ventilation at some point during their clinical course. Endotracheal intubation occurred predominantly before initiation of TH in 341 neonates (80%) and during hypothermia in 34 neonates (8%). 50 neonates (12%) were managed with CPAP during TH, and 3 neonates (1%) did not require any form of respiratory support. The median duration of respiratory support was 118 h (IQR 93–161). There was a statistically significant difference in length of hospital stay between the groups (*p* < 0.001). Neonates requiring invasive ventilation had a longer median hospital stay (18 days) compared with those treated exclusively with CPAP (12 days). Mortality was 13% in invasively ventilated neonates versus 0% in CPAP‐only neonates (*p* = 0.003). (Table 2).

**TABLE 1:** Baseline characteristics of neonates undergoing TH (*n* = 428). Data are presented as median with IQR or frequencies and percentages.

Characteristic	Overall cohort (*n* = 428)
Gestational age [weeks]	39 + 3 (IQR 38 + 1–40 + 4)
Birth weight [g]	3420 (IQR 2985–3750)
Apgar scores	
1 min	1 (IQR 1–3)
5 min	4 (IQR 2–5)
10 min	5 (IQR 3–7)
Initial pH	6.88 (IQR 6.8–6.96)
Base deficit [mmol/l]	−19 (IQR −22 to −16)
Lactate [mmol/l]	14 (IQR 10–18)
Resuscitation after delivery	97 (23%)
HIE severity (Sarnat score)	
Normal (Sarnat 0)	3 (1%)
Mild (Sarnat 1)	9 (2%)
Moderate (Sarnat 2)	171 (40%)
Severe (Sarnat 3)	107 (25%)
Initial aEEG background activity	
Continuous normal voltage	44 (10%)
Discontinuous normal voltage	132 (31%)
Burst supression	116 (27%)
Low voltage	40 (9%)
Flat trace	35 (8%)

**TABLE 2:** Outcome by ventilation mode in neonates with HIE.

Parameter	CPAP‐only (*n* = 50)	Invasive Vvntilation (*n* = 375)	*p*
Median hospital stay (days)	12	18	< 0.001
Mortality (%)	0	13	0.003

### 3.1 Sedation

Sedative and/or analgesic medication was administered in the majority of neonates included in the study (*n* = 406, 95%). Only sevem neonates (2%) did not receive any form of sedation during their clinical course; of these, three were supported with CPAP and four were invasively ventilated.

Sedation was more frequently used in invasively ventilated neonates compared with those managed with CPAP (88% vs. 11%, *p* = 0.08).

Among sedated neonates, commonly used agents included opioids and sedative drugs, in particular morphine (68%), fentanyl (21%), remifentanil (4%), sufentanil (8%), midazolam (31%), clonidine (21%), dexmedetomidine (13%) and ketamine (4%), administered either as monotherapy or in combination.

Morphine was more frequently administered in neonates supported with CPAP compared with those receiving invasive ventilation (80% vs. 67%, *p* = 0.09). Fentanyl was exclusively used in invasively ventilated neonates and not administered in infants supported with CPAP (24% vs. 0%, *p* < 0.001). Similarly, sufentanil was only used in invasively ventilated neonates (72% vs. 0%, *p* = 0.03). Clonidine was used with comparable frequency in CPAP‐supported and invasively ventilated neonates (82% vs. 85%, *p* = 0.78). Likewise, there was no relevant difference in the use of dexmedetomidine between groups (57% vs. 70%, *p* = 0.67).

Midazolam was more frequently administered in invasively ventilated neonates compared with those supported with CPAP (88% vs. 40%, *p* = 0.2).

### 3.2 Oxygen Requirements

During the first 6 h of life, neonates ventilated only with CPAP had significantly lower maximum FiO_2_ requirements than invasively ventilated infants (median 0.3 [IQR 0.21–0.50] vs. 0.5 [IQR 0.3–1.0], *p* < 0.001). Any oxygen demand, defined as FiO_2_ > 0.21 within the first 6 h of life, was more frequent in invasively ventilated neonates than in those supported with CPAP (31% vs. 13%, *p* = 0.01). Invasively ventilated neonates required supplemental oxygen for a longer duration than those managed with CPAP (median 4 days [IQR 1–7] vs. 1 day [IQR 0–2], *p* < 0.001).

### 3.3 pCO_2_ Patterns

Significant differences in pCO_2_ values were observed between ventilation modes. Within the first 6 h of life, invasively ventilated neonates exhibited higher maximum pCO_2_ levels compared with infants managed with CPAP (median 70.0 [IQR 46–95] vs. 55 [IQR 43–70] mmHg, *p* = 0.005). In contrast, minimum pCO_2_ values were higher in the CPAP group (36 [30–47] vs. 33 [25–41] mmHg, *p* = 0.02). Analysis of pCO_2_ values over the first 72 h of life confirmed persistently higher maximum pCO_2_ levels in invasively ventilated neonates compared with those supported with CPAP (median 70 [IQR 56–93] vs. 60 [IQR 49–69] mmHg, *p* < 0.001). Minimum pCO_2_ values during this period were higher in neonates managed with CPAP (median [IQR]: 33 [IQR 28–40] vs. 28 [IQR 24–34] mmHg, *p* < 0.001).

### 3.4 Subgroup Analysis by Sarnat Score

Among neonates with Sarnat 2 (*n* = 171, 40%), 83% (*n* = 142) received invasive ventilation, 16% (*n* = 27) received CPAP, and two neonates did not require respiratory support. CPAP‐supported neonates required fewer days of supplemental oxygen than invasively ventilated neonates (median [IQR]: 1 [0–2] vs. 3 [1–7] days; *p* < 0.001).

Blood gas analyses showed no significant differences in maximum or minimum pCO_2_ during the first 6 h or 72 h of life. Maximum FiO_2_ in the first 6 h was 0.35 [0.21–0.5] for CPAP and 0.45 [0.28–1.0] for ventilated neonates (*p* = 0.17). Breastfeeding was established earlier in CPAP‐supported neonates (median 5 days [IQR 4–6] vs. 7 days [5–10], *p* = 0.001) and had shorter hospital stays (median 12 days [10–14] vs. 15 days [12–19], *p* < 0.001). Mortality was 3% in the ventilated group and 0% in the CPAP group (*p* = 0.38).

Among neonates with Sarnat 3 (*n* = 107, 25%), 99% (*n* = 106) received invasive ventilation and 1% (*n* = 1) received CPAP.

### 3.5 Subgroup Analysis by aEEG Background Pattern

The distribution of aEEG background activity differed between ventilation groups. Among CPAP‐treated neonates, 30% had continuous normal voltage (CNV), 47% had discontinuous normal voltage (DNV), and 23% had burst suppression (BS), low voltage (LV), or flat trace (FT) patterns. Among invasively ventilated neonates, 10% had CNV, 35% had DNV, and 56% had BS, LV, or FT patterns.

Outcomes stratified by aEEG pattern are summarized in Table 3. In the CNV and DNV subgroups, CPAP‐supported neonates required fewer days of supplemental oxygen (CNV: 1 [0–4] vs. 1 [0–5], *p* = 0.13; DNV: 1 [0–1] vs. 2 [1–6.25], *p* < 0.001) (Figure 1) and started oral feeding earlier (CNV: 4 [3–8] vs. 5.5 [4–7.25] days, *p* = 0.26; DNV: 4 [4–6] vs. 7.5 [6–11] days, *p* < 0.001) compared with invasively ventilated neonates. Hospital stay was shorter in CPAP‐supported neonates in both subgroups (CNV: 12 [6–15] vs. 13.5 [10–16.5] days, *p* = 0.04; DNV: 12 [11–14] vs. 17 [12–21] days, *p* < 0.001).

**TABLE 3:** Clinical outcomes by aEEG background pattern and ventilation mode.

aEEG	Ventilation	*n*	O_2_ days	Max pCO_2_ 6 h (mmHg)	Min pCO_2_ 6 h (mmHg)	Max pCO_2_ 72 h (mmHg)	Min pCO_2_ 72 h (mmHg)	Time to oral feeding (days)	Hospital stay (days)
CNV	Vent	22	1 [0–5]	73.5 [56.5–92]	39.5 [27.5–47]	75.5 [65.5–93.5]	29 [24–32]	5.5 [4–7.25]	13.5 [10–16.5]
	CPAP	11	1 [0–4]	48 [42–59]	36 [31–39]	50 [42–61]	31 [28–36]	4 [3–8]	12 [6–15]
DNV	Vent	73	2 [1–7]	69 [45–89]	34 [26–40.5]	67 [54.5–85.5]	30 [24.5–36]	7 [6–11]	17 [12–21]
	CPAP	19	1 [0–1]	60 [43–80]	35 [29–51]	60 [56–69]	32 [28–44]	4 [4–6]	12 [11–14]
BS	Vent	51	5 [1–7]	80 [44–95]	33 [27–42]	80 [53–98]	30 [26–34]	8 [7–12]	18 [14–27]
	CPAP	1	—	—	—	—	—	—	—
LV	Vent	18	5.5 [1–10]	71.5 [46–85]	36.5 [31.5–44.5]	58.5 [51–73]	30.5 [27–35]	9 [5.75–12]	17 [13.5–22]
	CPAP	2	3.5 [3–3.5]	49.5 [40–49.5]	38.5 [36–38.5]	53.5 [48–53.5]	38 [36–38]	6.5 [5–6.5]	15.5 [15–15.5]
FT	Vent	12	7 [3–10.5]	76.5 [47–89]	28.5 [20.5–35]	64 [50–77]	26 [20.5–34]	11 [7–19]	24.5 [18–31.5]
	CPAP	1	—	—	—	—	—	—	—

*Note:* Data for the CPAP subgroups with BS and FT are not shown because each subgroup consisted of a single patient (*n* = 1), making summary statistics and statistical comparisons not meaningful.

**FIGURE 1:**
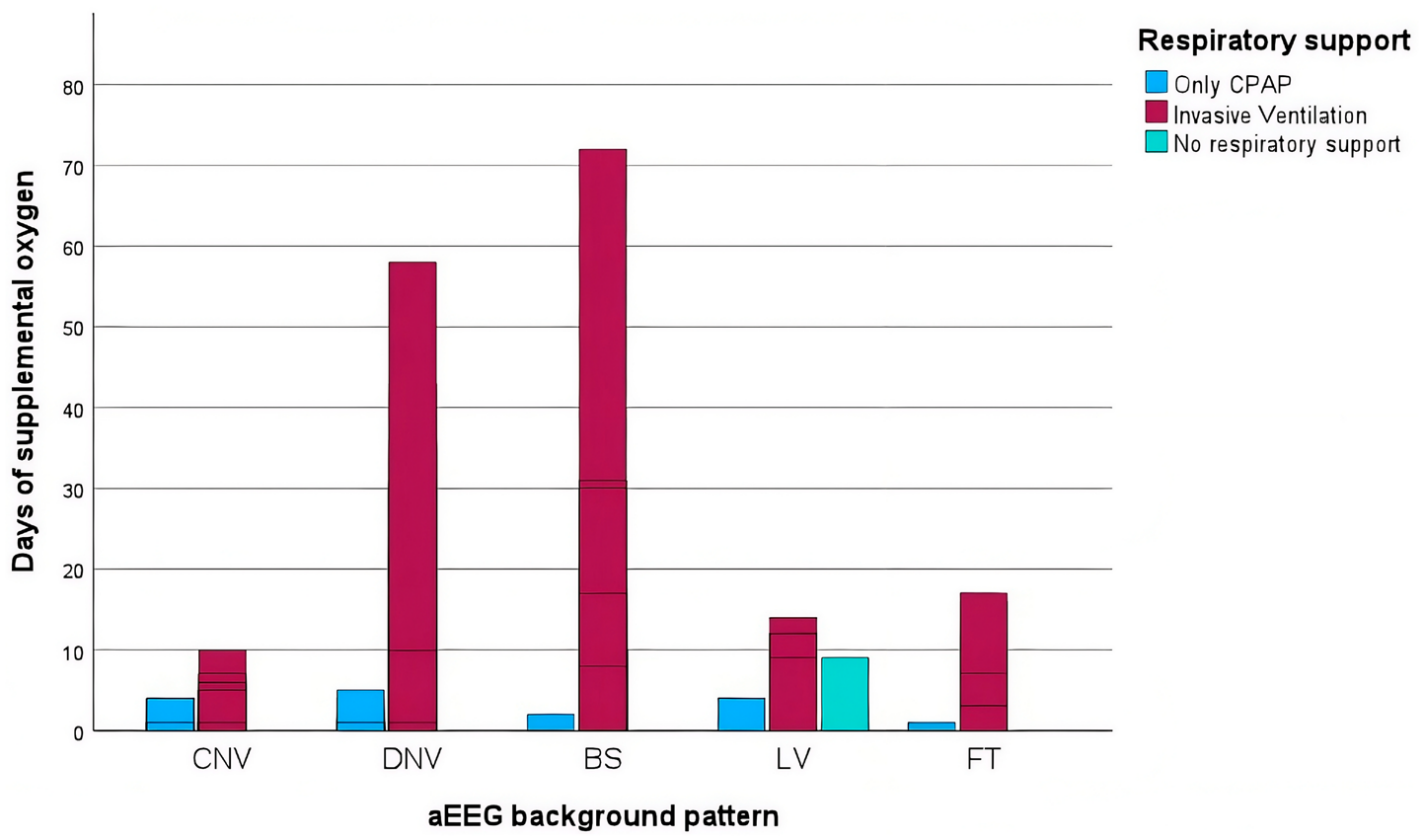
Oxygen supplementation duration by aEEG pattern and respiratory support in neonates undergoing TH.

In the BS, LV and FT subgroups, differences between CPAP and invasive ventilation were smaller, with some variation in supplemental oxygen days and hospital stay (BS: 0 [0–1] vs. 5 [1–7] days, *p* = 0.05; LV: 4 [3–4] vs. 4 [1–8.25] days, *p* = 0.87; FT: 0.5 [0–0.5] vs. 5 [1–8.5] days, *p* = 0.04) (Figure 1). Differences in time to start oral feeding and hospital stay in these subgroups did not reach statistical significance (Figure 2).

**FIGURE 2:**
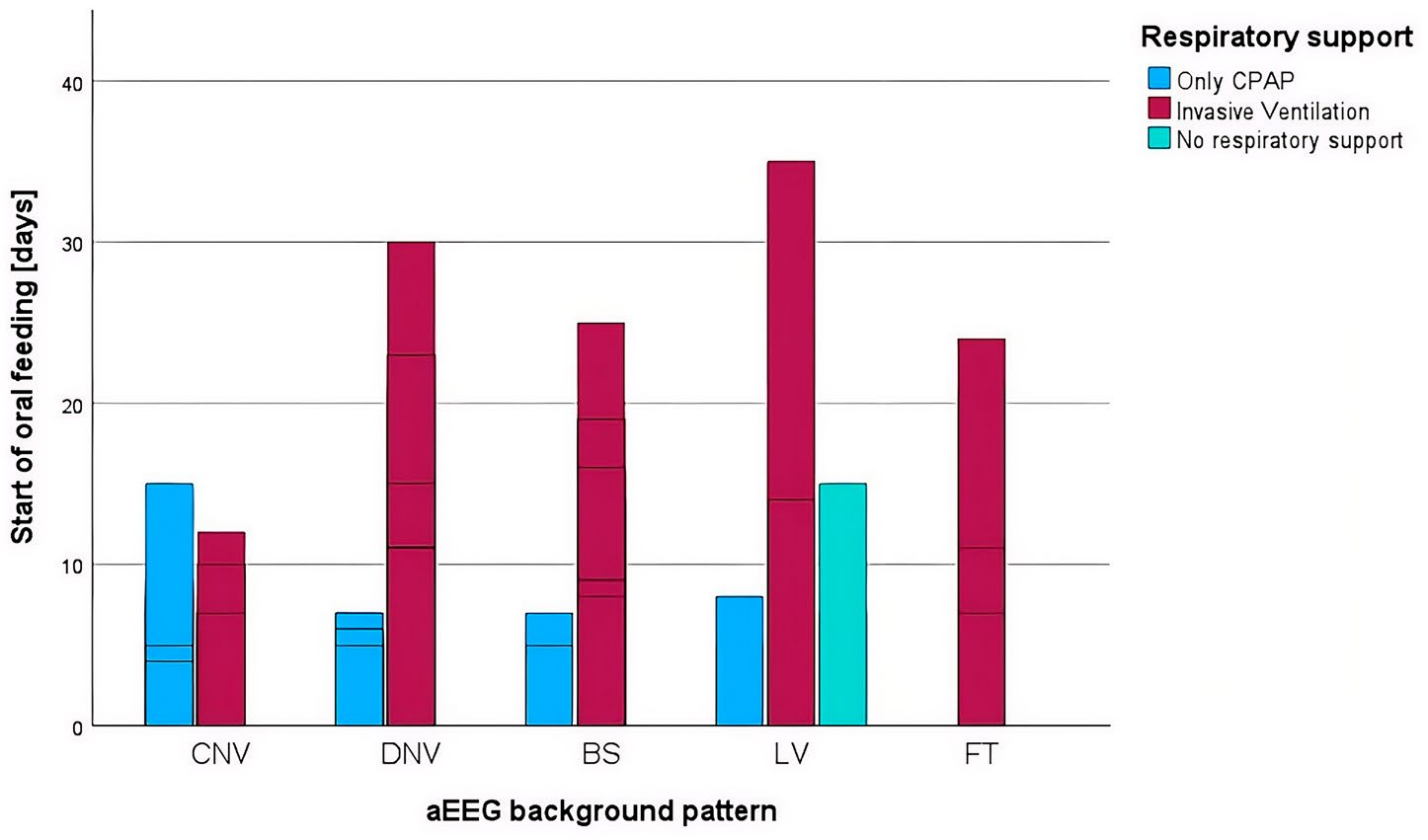
Time to initiation of oral feeding by aEEG pattern and respiratory support in neonates undergoing TH.

## 4 Discussion

This large multicentre registry‐based study demonstrates that respiratory management during TH in neonates with HIE is highly variable and associated with distinct and clinically relevant patterns of both pCO_2_ and oxygen exposure. While the majority of neonates required invasive ventilation at some point during their clinical course, a notable proportion were successfully managed with non‐invasive respiratory support, including neonates with moderate encephalopathy. Importantly, we identified strategy‐specific differences in gas exchange: invasively ventilated neonates exhibited higher maximum pCO_2_ levels, whereas infants supported with CPAP had higher minimum pCO_2_ values, suggesting a higher risk of severe hypocapnia. In parallel, invasively ventilated neonates required higher FiO_2_ and longer durations of oxygen supplementation. These findings indicate that respiratory support modality is not only a marker of disease severity but may also influence exposure to key physiological factors that are closely linked to secondary brain injury.

Analysing by encephalopathy severity revealed that the feasibility and benefits of non‐invasive support were greatest among neonates with moderate HIE (Sarnat 2). In this subgroup, CPAP‐supported infants required fewer days of supplemental oxygen, initiated enteral feeding earlier and had shorter hospital stays compared with invasively ventilated neonates, without significant differences in maximum or minimum pCO_2_ over the first 72 h. Conversely, nearly all neonates with severe HIE (Sarnat 3) required invasive ventilation, highlighting that disease severity remains the primary determinant of respiratory support in the most critically ill neonates.

Analyses based on aEEG background activity further refined these findings. Infants with favourable aEEG patterns, CNV or DNV, were more likely to tolerate CPAP and experienced improved short‐term outcomes, including reduced oxygen supplementation and earlier oral feeding. In contrast, neonates with severely abnormal aEEG patterns (burst suppression, low voltage, or flat trace patterns) showed minimal differences between CPAP and invasive ventilation, indicating that neurological severity limits the potential benefits of non‐invasive support. These findings indicate that aEEG may be a useful tool to guide the selection of respiratory support strategies during TH.

Regarding sedation, no significant differences were observed between the CPAP and invasive ventilation groups in the present study. Although sedation may effectively reduce stress responses and shivering [17] in particular and may lower cerebral metabolic activity, our analyses do not allow conclusions regarding its impact on neurological outcomes. In most cases, sedation was associated with mechanical ventilation carrying the risks of hypocapnia‐induced cerebral vasoconstriction and impaired cerebral perfusion, which may exacerbate brain injury. Moreover, sedation may contribute to hemodynamic instability and interfere with physiological autoregulation. Notably, infants with HIE have reduced clearance of sedative medications, such as morphine [18]. The ventilation‐dependent differences in pCO_2_ and oxygen exposure found in the present study are physiologically plausible. Invasive ventilation during TH may predispose to hypercapnia due to impaired respiratory drive, sedation, and challenges in ventilator adjustment under hypothermia. Conversely, CPAP preserves spontaneous breathing and may reduce the risk of iatrogenic hypocapnia, although compensatory hyperventilation in response to metabolic acidosis remains possible. Both strategies carry characteristic risks for CO_2_ instability, underscoring the need for individualized monitoring.

An additional consideration when interpreting the observed pCO_2_ differences is that blood gas values were reported as measured, and it remains unknown whether temperature correction was applied during TH. Non‐temperature‐corrected pCO_2_ values generally underestimate the physiological effect of CO_2_ at reduced body temperatures and may therefore not accurately reflect the actual cerebral exposure to CO_2_ [19]. This issue may be particularly relevant when comparing CPAP‐supported and invasively ventilated infants, as the physiological mechanisms governing CO_2_ regulation differ between these groups. Infants receiving CPAP maintain spontaneous respiratory effort and may exhibit variable compensatory responses to metabolic changes, whereas mechanically ventilated infants are more dependent on ventilator settings and sedation‐related effects on respiratory control. Consequently, systematic differences in CO_2_ handling between respiratory support strategies may have resulted in either underestimation or overestimation of the observed intergroup differences in pCO_2_ patterns. Similarly, the higher FiO_2_ requirements and prolonged oxygen exposure observed in invasively ventilated infants may increase the risk of oxidative stress during reperfusion, which has been implicated in secondary neuronal injury.

Beyond the choice of ventilation mode, our findings raise the question of how precisely oxygenation and ventilation targets can be maintained in routine clinical practice. Both oxygen exposure and pCO_2_ levels showed substantial variability, despite the well‐established importance of avoiding hypo‐ and hyperoxia as well as hypo‐ and hypercapnia in this vulnerable population. In this context, emerging technologies such as automated FiO_2_ control systems and closed‐loop ventilation strategies may offer a promising approach to reduce variability in gas exchange. While these systems have been shown to improve time within target ranges in other neonatal populations, data in neonates with HIE undergoing TH remain limited.

Importantly, the feasibility of tight manual control of FiO_2_ and ventilation parameters may be challenged by increasing workload and staffing constraints in NICUs. Particularly in invasively ventilated infants, maintaining stable gas exchange requires frequent adjustments and continuous monitoring, which may not always be achievable in routine care.

Our findings have several clinically relevant implications. First, non‐invasive support is feasible in selected neonates with moderate HIE and favourable aEEG, offering the potential to reduce sedation, facilitate earlier enteral nutrition and shorten hospital stay. Second, strategy‐specific CO_2_ and oxygen patterns highlight that respiratory management during TH is an active component of neuroprotective care rather than a purely supportive intervention. Frequent monitoring of arterial or transcutaneous CO_2_ and careful titration of oxygen are essential to maintain cerebral perfusion and minimize oxidative injury. Third, the presence of preserved spontaneous breathing across a broader spectrum of disease severity challenges the assumption that CPAP is only appropriate in mild HIE. This raises important clinical questions regarding the optimal timing and selection criteria for intubation in cooled neonates. While some infants may ultimately require escalation of respiratory support, routine early intubation in all non‐mild cases may expose a substantial proportion of neonates to unnecessary invasive ventilation without clear evidence of benefit.

Previous studies have documented the frequent need for mechanical ventilation in HIE and its association with disease severity [1]. Our results extend these observations by demonstrating that even among neonates with comparable encephalopathy grades, ventilation strategy influences early clinical outcomes and exposure to physiologically relevant parameters. These findings align with prior reports linking early hypocapnia and hypercapnia to adverse neurological outcomes and emphasize the need for individualized ventilatory management during TH [15, 20, 21, 22].

Limitations of our study include its retrospective design and reliance on registry data, which limits assessment of continuous CO_2_ trends. Additionally, long‐term neurological outcomes were not included, and prospective studies are needed to link specific CO_2_ and oxygen exposure patterns to clinical outcomes. Additionally, blood gas values, including pCO_2_, were reported as measured, and it is unknown whether temperature correction was applied. As non–temperature‐corrected values tend to underestimate the physiological effect of CO_2_, the absolute values may not accurately reflect arterial CO_2_ at the neonates' actual body temperature, potentially affecting comparisons between groups.

## 5 Conclusion

In conclusion, our study demonstrates that respiratory management in cooled neonates with HIE is associated with strategy‐specific differences in CO_2_ and oxygen exposure, which may have important implications for cerebral perfusion and secondary brain injury. CPAP is feasible and safe in selected neonates with moderate HIE or favourable aEEG patterns, offering shorter oxygen supplementation, earlier enteral feeding and reduced hospital stay. These findings support an individualized approach to respiratory support, where careful selection and monitoring can optimize short‐term clinical outcomes while minimizing the risks associated with mechanical ventilation. Prospective studies linking respiratory management, gas exchange patterns and long‐term neurological outcomes are essential to further optimize supportive care for neonates with HIE.

## Author Contributions

**Anne Groteklaes:** data curation, formal analysis, project administration, resources, writing – review and editing. **Bettina Bohnhorst:** writing – review and editing. **Hemmen Sabir:** conceptualization, data curation, methodology, project administration, resources, software, supervision, validation, writing – review and editing. **Kirsten Glaser:** writing – review and editing. **Andreas Müller:** resources, writing – review and editing. **Till Dresbach:** conceptualization, data curation, methodology, project administration, resources, software, supervision, validation, writing – review and editing. **Sebiha Demir:** conceptualization, data curation, formal analysis, methodology, project administration, resources, software, writing – original draft.

## Funding

The authors have nothing to report.

## Conflicts of Interest

The authors declare no conflicts of interest.

## Supporting information


**Appendix S1:** Members of the Hypothermia Registry Group Description. This table lists the contributing study group members and their respective hospitals who participate in the German Hypothermia Registry.

## Data Availability

The data that support the findings of this study are available from the corresponding author upon reasonable request.
